# Supplementary material for: FGL2/FcγRIIB Signalling Mediates Arterial Shear Stress-Mediated Endothelial Cell Apoptosis: Implications for Coronary Artery Bypass Vein Graft Pathogenesis
Source: Int J Mol Sci. 2024 Jul 11;25(14):7638. doi: 10.3390/ijms25147638 (PMC11277082; doi:10.3390/ijms25147638)
Supplement: Supplementary file 1 [file ijms-25-07638-s001.zip › ijms-2981274-supplementary.pdf]

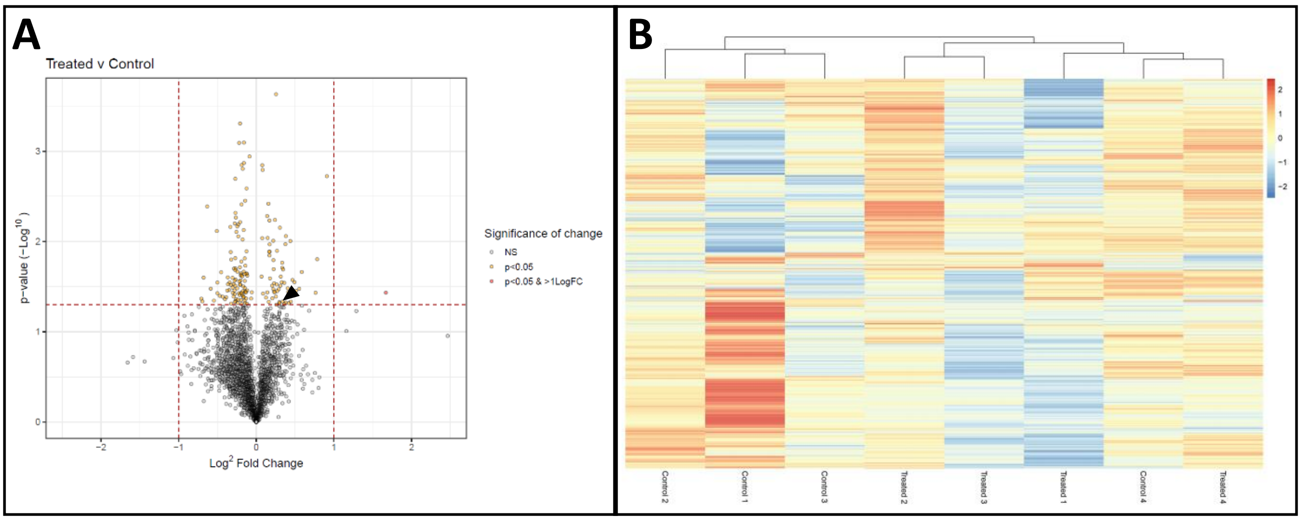

Figure S1: Tandem mass tagging proteomics.
HUVECs were pre-conditioned to venous FSS for 18h and exposed to venous or arterial FSS for 24h (n=4). Conditioned media was collected and concentrated, denatured and depleted of albumin prior to proteomic analysis. (A) Volcano plot (with FGL2 indicated by black arrow) and (B) Heat map of proteomic analysis.

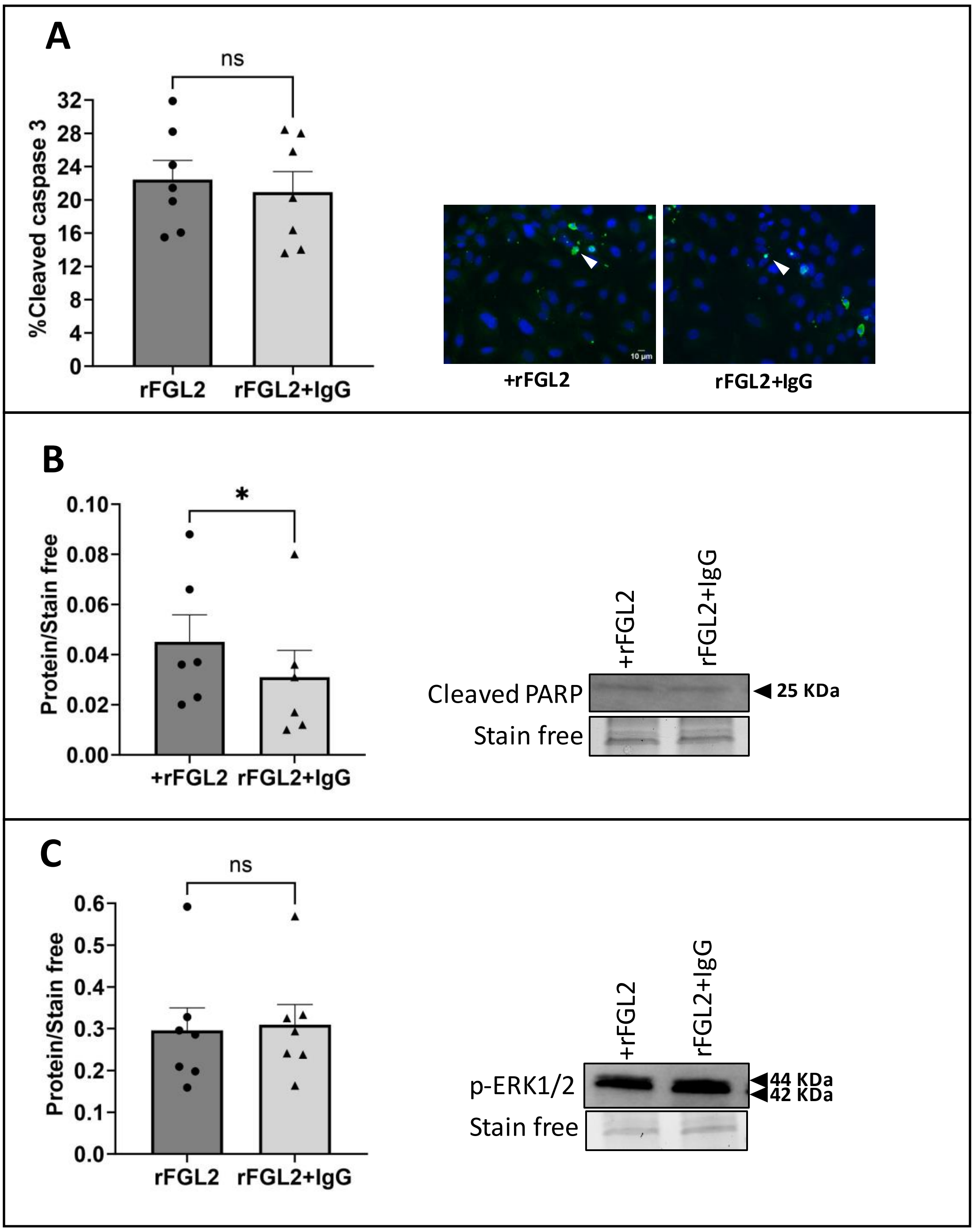

Figure S2: The effect of IgG on FcγRIIB activation.
*Static HUVECs were pre-incubated with a goat IgG control (2.5 µg/mL) for 45 minutes prior to treatment with rFGL2 (20 ng/mL) for 24h. The effect of IgG interaction with static HUVECs on endothelial apoptosis was quantified by (A) cells positive for CC3 immunocytochemistry (some indicated by white arrow; n=7; two-tailed, paired t-test) and (B) Western blotting for cleaved PARP (representative Western blot and stain free gel loading control; molecular weight indicated by black arrow; n=6; two-tailed, paired t-test). (C) Static HUVECs were likewise incubated with a goat IgG control and the effect of IgG binding FcγRIIB on ERK1/2 phosphorylation was quantified by Western blotting (representative Western blot and stain free gel loading control; molecular weight indicated by black arrow; n=7; two-tailed, paired t-test). Values expressed as mean ± SEM. * P<0.05.*

Gene name	Forward sequence 5’-	Reverse sequence 5’-
Intercellular adhesion molecule-1 (ICAM-1)	ACCATCTACAGCTTTCCG	TCACACTTCACTGTCACC
NFκB p65	AGGTGCAGAAAGAGGACATTGAGGT	AATGGCCACTTGTCGGTGCACATC
Fibroleukin (FGL2)	AGCTAAAGAATGCCAAAGAG	ATTAGATGTTGAACTGGACG
FcγRIIB	CAATCCCACTAATCCTGATG	CAATGGAGACTAAATACGGTTC

Table S1: Primers used in RT-qPCR experiments

Step	Sub-step	Temperature (°C)	Time
**Pre-incubation**		95	5 min
**Amplification – 45 cycles**	*Denaturing*	95	10 sec
	Annealing	60	10 sec
	Elongation	72	10 sec
**Melting**		95	5 sec
		65	1 min
		97	Continuous
**Cooling**		40	-1.5 °C/sec

Table S2: RNA RT-qPCR program
